# Bankruptcy Cascades in Interbank Markets

**DOI:** 10.1371/journal.pone.0052749

**Published:** 2012-12-31

**Authors:** Gabriele Tedeschi, Amin Mazloumian, Mauro Gallegati, Dirk Helbing

**Affiliations:** 1 Department of Economics, Universitá Politecnica delle Marche, Ancona, Italy; 2 Department of Humanities and Social Sciences, ETH Zurich, Zurich, Switzerland; MIT, United States of America

## Abstract

We study a credit network and, in particular, an interbank system with an agent-based model. To understand the relationship between business cycles and cascades of bankruptcies, we model a three-sector economy with goods, credit and interbank market. In the interbank market, the participating banks share the risk of bad debits, which may potentially spread a bank’s liquidity problems through the network of banks. Our agent-based model sheds light on the correlation between bankruptcy cascades and the endogenous economic cycle of booms and recessions. It also demonstrates the serious trade-off between, on the one hand, reducing risks of individual banks by sharing them and, on the other hand, creating systemic risks through credit-related interlinkages of banks. As a result of our study, the dynamics underlying the meltdown of financial markets in 2008 becomes much better understandable.

## Introduction

As economic literature has taught us in more than one occasion, there are many economic examples of situations in which mainstream theory, i.e., the Arrow-Debreu general equilibrium model, does not explain interactions between economic agents well. In particular, we believe that if we want to understand the dynamics of interactive market processes, and the emergent properties of the evolving market structures, it might pay to analyze explicitly how agents interact with each other, how information spreads through the market and how adjustments in disequilibrium take place.

To model how the agents’ decisions are influenced by their mutual interactions and the repercussions that these may have on the economic system, we use a “communication structure” based on network theory, in which nodes can represent agents and edges connective links measuring the intensity of interaction between agents.

The recent vicissitudes of the credit market are a natural research issue to be analyzed with graph theory. If the banks were “isolated units”, the bankruptcy of a borrower would be almost unimportant in the credit system. However, given the strong interdependence in the interbank market, the default of one bank can bring about phenomena of financial contagion.

In the last thirty years, in most advanced and developing economies, the financial sector has assumed an increasing relevance with respect to the production sector; furthermore, the role of the banking system has gradually shifted from the loan based financing of non-financial corporations to more market-based activities and speculative operations. This deep transformation, usually named as financialization of the economy, has not only increased the interdependence among financial institutions, but also determined an increase of “easy credit”. This has created asset bubbles and debt-induced economic booms, with the consequent rising of corporate debt-equity ratios and bank leverage that have made the economy increasingly fragile and potentially unstable. Following the severe financial and economic crisis that started in 2007 in US, the phenomenon of growing financialization is increasingly under critical discussion as some of the major causes of the crisis. Although different important interpretations of the current crisis have been proposed (see, for instance, [1]), the effect of the increasing globalization and financialisation of the economic system is, certainly, one of the key elements to understand the current crisis.

Three types of propagation of systematic failure have been studied in the literature. First, the bank runs, known as self-fulfilling panic [2]–[6]. Second, the asset price contagion [7], [8]. Third, the inter-locking exposures among financial institutions [8]–[13].

Following this last line of research, in this paper we are explicitly concerned with the potential of the interbank market to act as a contagion mechanism for liquidity crises and to determine macroeconomics outcomes such as bankruptcies. Allen and Gale (2000), Thurner et al. (2003) and Iori et al. (2006) have shown that, modeling the credit system as a random graph, when increasing the degree of connectivity of the network, the probability of bankruptcy avalanches decreases. However, when the credit network is completely connected, these authors have proven that the probability of bankruptcy cascades goes to zero. The explanation for this result is that, in credit networks, two opposite effects interact. On the one hand, increasing the network connectivity decreases the banks’ risk, thanks to risk sharing. On the other hand, increasing the connectivity rises the systemic risk, due to the higher numbers of connected agents which, in case of default, may be compromised. According to the three cited models, the impact of the risk sharing plays a leading role. So, in these models there is a benefit in creating links between agents, because they allow to diversify risk.

An exception to this view is the recent contribution by Lorenz and Battiston (2008), where the authors show that the introduction of a trend reinforcement in the stochastic process, describing the fragility of the nodes, generates a trade-off. Rising the connectivity, the network is less exposed to systemic risk, in the beginning, thanks to risk sharing. However, when the connectivity becomes too high, the systematic risk eventually increases.

A forerunner of this trade-off between risk sharing and systemic risk was already present by Iori et al. (2006), where the authors showed that, in the presence of heterogeneity, a non-monotonic relationship between connectivity and systemic risk exists.

In the present paper, we deal with the correlation between risk sharing and connectivity in the interbank system. In view of the recent economic crisis, in fact, the linear relationship between connectivity and systemic risk should be reassessed. Spreading the risk around the globe may indeed improve stability in good times thanks to risk sharing. However, in times of crisis, we believe that the effect of critical perturbations can spread across the whole system. Therefore, the credit market as a network with interdependent units, is exposed to the risk of joint failures of a significant fraction of the system, which may create a domino effect such as bankruptcy cascades.

A recent model that is related to ours is that of Battiston et al. (2012a). The authors show that, in the presence of financial acceleration - i.e., when variations in the level of financial robustness of institutions tend to persist in time or to get amplified - the probability of default does not decrease monotonically with connectivity. Along this line, several authors have started to analyze the correlation between connectivity and probability of bankruptcy in credit networks. Many theoretical studies have found a non-monotonic relationship between these two variables. In particular, many recent models [14], [15] have shown that the diversification of credit risk across many agents has ambiguous effects on systemic risk.

The problems arising from financial market interconnectedness have also been highlighted by empirical studies which have emphasized structural properties of lending networks before and after the current financial crisis [16]–[20] and defined new analytical tools able to better identify and monitor systemic risk and crisis transmission[21]–[23].

Our model represents a simple three-sector economic system (considering goods, credit and an interbank market), involving firms and banks. Two types of credit are considered: loan and interbank credit. According to the economic situation, companies may ask for money from financial institutions to increase their output. In this case, firms enter the credit market and consult with a fixed number of randomly chosen banks. Banks consider the investment risk and finally decide whether to offer the requested loan and define interest rates. After this first consultation meeting, each firm asks the banks it links with for credit, starting with the one with the lowest interest rate. If this bank faces liquidity shortage when trying to cover the firms’ requirements, it may borrow from a surplus bank.

In the interbank market, we assume a random connectivity among banks. If one or more firms are not able to pay back their debts to the bank, the bank’s balance sheet decreases. To improve its own situation, the bank rises the interest rate offered to other firms, eventually causing other defaults among firms. The bad debt of companies, affecting the equity of financial institutions, can lead to bank failures as well. Since banks, in case of shortage of liquidity, may enter the interbank market, the failure of borrower banks could lead to failures of lender banks. The interest rate, thus, can bring about a cascade of bankruptcies among banks. The source of the domino effect may, on one side, be due to indirect interactions between bankrupt firms and their lending banks through the credit market and, on the other side, due to direct interactions between lender and borrower banks through the interbank system.

The originality of this work compared to Battiston et al. (2012a) is the introduction of three interacting markets influencing each other. In this way, we can study the impact of systemic risk not only on the agents’ dynamics such as their financial fragility, but also on the business cycle and economic growth. In this regard, we study the effect of an exogenous shock on a specific firm by increasing the connectivity in the interbank system, and we observe that the systemic risk prevails over the advantages of risk sharing. Although the demand of loans and the number of granted loans stay almost the same by changing the connectivity in the inter-bank system, surprisingly, with higher connectivity we observe larger cascades of bankruptcies among banks. As shown in Iori et al. (2006), we find that the root of avalanches lies in the agents’ heterogeneity. In particular, our results show that the degree of contagion depends on the size of losses imposed by failing debtor banks on creditor banks in the system (see [24]–[27] for empirical analysis). Moreover, in line with other works [28], [29], we show that financial crises are characterized by the procyclicality of leverage across financial institutions.

Furthermore, we also find that the holding of large liquid reserves, while generally stabilizing in the interbank market, reduces the growth of aggregate output by decreasing granted loans and therefore firm investments.

The remainder of the paper is organized as follows. First, we describe the model with the behavior of firms and banks. Then, we discuss the results of computer simulations for the baseline model and for the model with the interbank system. Finally, the last section presents conclusions.

## Structure of the Model

Our model represents a three-sector economy: goods, credit and the interbank market.

We consider a sequential economy populated by a large number of firms 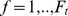 and banks 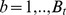, which undertake decisions at discrete time, denoted by *t = 0,1,2,…,T*.

In the goods market, output is demand-driven, that is firms, given their production constraints, sell as much output as the market can absorb. However, incomplete information about the market potential can generate a gap between the firms’ expected and realized demand. In this disequilibrium scenario, supply does not (necessarily) match aggregate demand, so the goods market may be out of equilibrium. In this way, the model is able to generate an unexpected shock to the revenues of firms, so that their profit may become negative.

To meet their expected demand, companies make investments using the credit market. Therefore, in each time period, a subset of firms enter in the credit market asking for credit. The amount of credit requested by firms is related to their investment expenditure, which is therefore dependent on their expected demand, interest rate and firm’s economic situation.

The primary function of banks activity is to lend their funds through loans to firms, as this is their way to make money via interest rates. Banks consulted by companies, after analyzing their credit risk, may grant the requested loan, when they have enough supply of liquidity. However, since banks adopt a system of risk management based upon an equity ratio, companies may not receive requested loans even if banks have enough supply of liquidity. If consulted banks do not have liquidity to lend, they can enter the interbank market, in order not to lose the opportunity of earning on investing firms. The interbank market has the same structure as the credit market.

### Firms

In each time period *t*, we have a large finite population of competitive firms indexed by 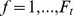. The overall population 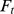 of firms is time dependent because of endogenous entry and exit processes to be described below. Firms are profit seekers. Therefore, at any time period *t*, they try to maximize their expected profits, by forecasting the market demand.

Following some of the key elements of behavioral agent-based models, closely related to Keynes’ view that ‘expectations matter’, to Simon’s view that economic man is boundedly rational and to the view of Kahneman and Tversky that individual behavior under uncertainty can best be described by simple heuristics and biases [30]–[34], we model a gap between a firm’s actual demand 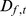 and its expected demand 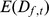. Demand 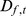 is defined as
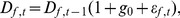
(1)where 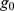 is a constant, 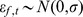 is a normally distributed variable and the expected demand is 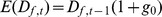.

To produce a homogeneous output 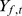, the firm *f* uses its capital 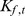 as the only input. The firm’s production function is
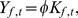
(2)where the capital productivity 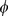 is assumed to be constant and uniform across firms for simplicity. However, given the incomplete information about the demand, firm *f* decides to produce as much as it expects the market to be able to absorb. In this light, the production function mirrors the maximum output that firm *f* can produce at any time *t*. This amount, however, can shrink due to a lack of the expected demand.

To clarify, assume that 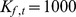 and 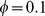. This means that the firm can produce up to 100 goods. If its expected demand 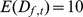, it will just produce 10, as it is the maximum amount that the company forecasts to be able to sell. However, if its expected demand is 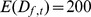, the firm will produce 100, as it cannot produce more with its capital. In the latter case, the firm will ask for a loan from the credit market to increase its productivity and satisfy expected demand in the future.

The only external source of finance that firms have is the loan from banks [35], [36]. The firm’s demand of loan to reach the expected demand is
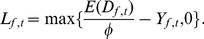
(3)


Eq. (3) reproduces an empirical evidence: lending often increases significantly during business cycle expansions, and then falls considerably during subsequent downturns [37], [38]. Consistent with this stylized fact, Federal Reserve Chairman Alan Greenspan (Chicago Bank Structure Conference, May 10, 2001) noted that at the bottom of the cycle, ``the problem is not making bad loans […] it is not making any loans, whether good or bad, to credit-worthy customers”, consistent with the sometimes dramatic fall in lending during cyclical downturns [39]–[41]. Eq. (3) therefore should be interpreted as a new micro-foundation, and its relevance and reliability is grounded by empirical evidence. Nevertheless, since borrowing is risky, the company considers its probability of bankruptcy and its risk aversion (see, for instance [42], [43]). To incorporate these elements into the model, we assume that the firm adjusts its demand of loan according to:
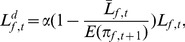
(4)where 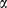 is a constant which mirrors the risk aversion coefficient and may be higher, lower, or equal to one, reflecting risk lover, adverse, and neutral respectively and 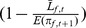 reflects the firm’s financial fragility based upon its debt commitments 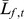 and expected profit 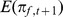 ratio. If firm *f* expects its next profit not to be enough to pay back its installments, it will ask for less loan.

At each time *t*, the debt commitments 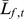 (interest & installment) for the firm *f* are 
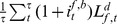
, where 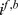 is the real interest rate that firm *f* pays to bank *b*. We assume that a loan given at time *t* to the firm *f* has to be payed back by the next 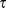 periods.

For simplicity, we furthermore assume that each firm has total variable costs equal to financing costs. Therefore, profits in real term are
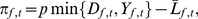
(5)where the selling price of one good is set to 1. Assuming that all the profits are retained [36], the firm’s capital stock changes are updated according to
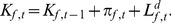
(6)


### Banks

Similar to companies, we have a time dependent finite population of competitive banks indexed with 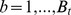.

When a firm needs loan, it contacts a number of randomly chosen banks. This means that a firm knows the credit conditions of few banks in each time step. Each contacted bank is assumed to offer an interest rate of
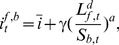
(7)where 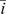 is set by the Central Bank and 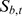 is the supply of liquidity of bank *b*. So the interest rate is decreasing as the bank’s financial robustness.

After exploring the lending conditions of the contacted banks, each firm asks the consulted banks for credit starting with the one offering the lowest interest rate. Banks deal with firms in a “first come, first served” basis. If a firm asks for a loan from a bank, either it receives the complete amount of the requested loan or it receives no money (where the bank may use the interbank market or not).

The regulation of financial intermediaries (Basel I and II) forces banks to hold a capital caution of 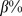 of liquidity to prevent bankruptcies due to unexpected losses. For the sake of simplicity, we model this regulatory parameter assuming that banks give the requested loan with a certain probability.
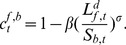
(8)


This means, for example, out of 10 different requested loans with 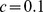, one loan will be given. By increasing 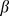, banks are forced to hold in reserve a larger percentage of their liquidity. 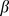 has to be interpreted as the fraction of risk that a bank is allowed to take within a given time step, as compared to its own liquidity. This threshold may be viewed as a regulatory parameter, since it imposes an upper limit for a bank’s risk dependent on its cash. It is a helpful tool to limit the bank’s risk, in particular the credit risk. Moreover, according to Eq. (8), the volume of credit given by a bank is proportional to its present liquidity. The smaller the bank the smaller its transactions.

If the bank regulatory parameter is satisfied and the bank has enough supply of liquidity, then it grants the requested loan.

If the contacted bank has not enough supply of liquidity to fully satisfy the firm’s loan, then the bank considers to use the interbank market. Our goal is to understand how the interbank structure can influence the economic cycle and the bankruptcy among banks. As in the credit market, the requiring bank asks the lacking fraction of the loan requested by the firm from *x* randomly chosen banks. Among the contacted banks, the banks satisfying the risk threshold in Eq. (8) and having enough supply of liquidity offer the loan to the asking bank for an interbank interest rate, which equals the credit market interest rate in Eq. (7). Among this subset of offering banks, the bank 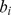 (borrower) chooses the bank, starting with the one offering the lowest interest rate. When it receives the requested loan, the bank lend it to the asking firm.

Bank supply of liquidity 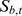, evolves according to:
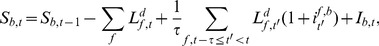
(9)where the second term (right side) shows the total loan of bank *b* at time *t*, the third term denotes the installment and the interest that the bank receives from the ‘safe’ firms, to which it has given a loan not before 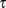 time steps ago, and the last term, 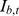, reflects the lending by bank *b* from other banks at time *t*. Note that 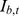 can be negative or positive, depending on whether the bank is creditor or debtor. In case of interbank borrowing, as for the firms, interests and installments must be paid back within the next 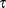 periods. When, for instance, we consider the borrower bank 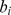, 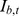 is
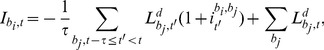
(10)where 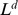 is the credit that the bank 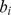 obtain from 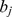. It is important to underline that 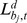 is immediately used by 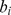 to lent firm f.

Like companies, banks are profit seekers. A bank’s profits in time *t* is:
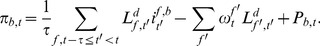
(11)


The bank’s profit depends on the interests payed by firms (first term), on the firms’ bad debt, with 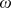 to be the share of loan that firms could not pay back because they went bankrupt (second term) and on the interbank credit (third term). Note that 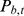 is positive if the bank lends in the interbank system, otherwise zero. Considering the lending bank 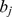, 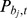 is
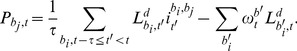
(12)


As in Eq (11), the first term mirrors interests payed by debtor banks and the second term is the banks’ bad debt (losses).

### Bankruptcy Conditions and Demography of Firms and Banks

Because of the uncertain environment, agents may go bankrupt. In this model, bankruptcy happens to firms or banks when they do not have enough ‘cash’ (revenues) to pay their loans back. In this sense, we are much closer to the idea of liquidity crisis than to the financial fragility conditions of Greenwald and Stiglitz framework. When agents go bankrupt, they leave the market. We also assume that an agents leave the market if it fails to receive requested loans for *s* consecutive time steps.

Regarding entries, we follow the approach of Delli Gatti et al. (2005). The economic literature has suggested models ranging from exogenously stochastic processes [44], where authors assume a simple mechanism of entrance based on a one-to-one replacement, to models with an endogenous entry process, which depends on expected profit opportunities [45], [46]. These last theories argue that the entrance of new firms in an industry will be influenced by the amount of sunk costs in the sector. A greater degree of sunk costs should reduce the likelihood of entry (see [47], [48] for empirical evidence).

Our modeling strategy aims at reproducing this evidence. The number of new entrants (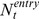) is obtained by multiplying a constant 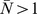 with a probability, which depends negatively in the case of firms and positively in the case of banks on the average lending interest rate:
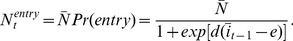
(13)where *d* and *e* are constants. The higher is the interest rate, the higher are firms debt commitments, and the lower (higher for banks’ side) are expected profits, with entries being lower (higher for banks’ side) in number.

Moreover, in line with the empirical literature on firm entry ([49]; [50]), we assume that entrants are on average smaller than incumbents, with the stock of capital of new firms and the supply of liquidity of new banks being a fraction of the average stocks of the incumbents. So, entrants’ size in terms of their capital stock is drawn from a uniform distribution centered around the mode of the size distribution of incumbent firms/banks.

## Simulation Results

We explore the dynamic properties of the economic system modelled above by means of computer simulations. We consider an economy initially consisting of 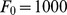 firms and 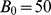 banks and study it over a time span of 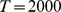 periods. Each firm is initially given the same amount of capital 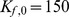 and demand 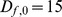. We fix 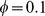, 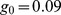, 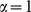, 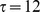. Firm entrance parameters are 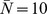, 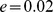, and 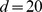.

Each bank is initially given the same amount of liquidity 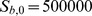. We fix the Central Bank interest rate 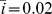, 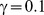, 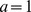, 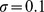, and 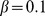. Despite the homogeneous initial conditions, the economy develops heterogeneous distributions through the interaction of noise and feedback effects.

In order to get rid of transients we evaluate only the last 1600 simulated periods. Simulations are repeated 100 times with different random seeds.

### Stylized Facts of the Benchmark Model

Let we start from a sort of “benchmark” setup, for which the model jointly accounts for an ensemble of stylized facts regarding both “micro/meso” aggregates such as indicators of industrial structures (e.g. firm size distributions and firm growth rates) together with macro statistical properties (including rates of output growth and output volatility).

**Figure 1:**
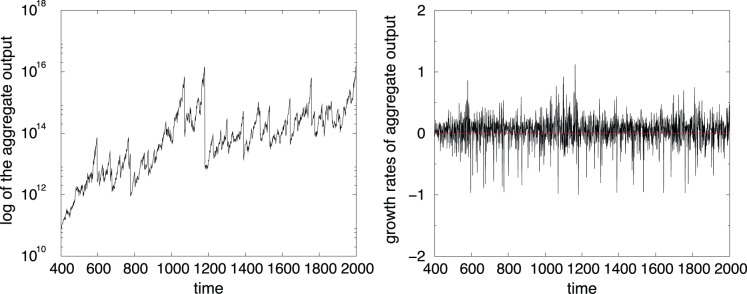
Evolution of the aggregate output (left side) and growth rates of the aggregate output (right side), as a function of time.

First of all, the model robustly generates endogenous self-sustained growth patterns characterized by the presence of persistent fluctuations, as shown in Figure 1 (left side).

Indeed, aggregate fluctuations, measured by output growth rates (right side of Figure 1), are path dependent (i.e., nominal shocks have real and permanent effects). Moreover, they are characterized by cluster volatility, a well-known property in the financial literature (see for instance [51]). This implies that large changes in variable values tend to cluster together, resulting in a persistence in the amplitudes of these changes. A quantitative manifestation of this fact is that, absolute growth rates display a positive, significant and slowly decaying autocorrelation function. In our case, the autocorrelation parameter is equal to 0.95, a value very close to that found for the quarterly empirical data for the G7 countries, which is 0.93 [52].

**Figure 2:**
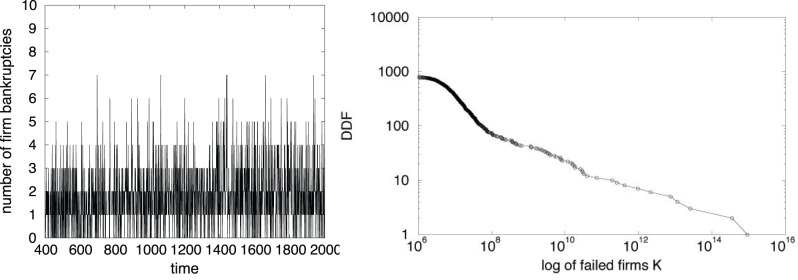
Time evolution of firm bankruptcies (left side) and decumulative distribution function of failed firms’ size (right side).

In addition to fluctuations resembling business cycles, the simulated time path of aggregate activity is characterized by a broken-trend behavior. The model is able to generate an alternation of aggregate booms and recessions as a non-linear combination of idiosyncratic shocks affecting individual decision-making processes. The account of business cycles offered by the agent based model thus contrasts sharply with DSGE theory, according to which fluctuations in aggregate activity are explained by random variations in aggregate TFP growth. In our simulations, depressions are due to the failure of big firms. Indeed, since we do not impose any aggregate equilibrium relationship between the firms actual demand and their expected demand, our simulated market generates individual out-of-equilibrium dynamics. Due to the absence of any exogenously imposed market-clearing mechanism, the economy is allowed to self-organize towards a spontaneous order with persistent excess demands, which have important consequences on the dynamic of firms. In fact, the gap between the expected and actual demand may generate an unexpected shock to firms’ profits, able to trigger bankruptcies of firms. If one or more companies are not able to pay back their debts to banks, then also banks suffer with a decrease in their equity level. Consequently, in order to improve their own situation, banks rise the interest rate to all the firms in their portfolio, eventually causing other defaults among companies. Figure 2 (left side) displays the time series of firm defaults, which are roughly constant during the simulation even when the system experiences severe breakdowns. This feature of the model underlines the important role of heterogeneity. In fact, in Figure 2 (right side), we show that crises do not depend on the quantity of bankrupted agents, but on their ‘quality’. The same economic process can thus produce small or large recessions depending to the size of failed companies.

In addition, it is important to note that the model provides an useful tool to predict crises. In line with Minsky’s Financial Instability Hypothesis (1992), we show that over periods of prolonged prosperity and optimism about future prospects, financial institutions grant more loans without considering borrowers financial fragility. A natural way to assess the co-movement between the increase (decrease) in aggregate output and increase (decrease) in the number of granted loans is to study their correlation. The Pearson correlation coefficient significant at 1% level between positive aggregate output changes and the number of granted loan reaches a value above 0.63, confirming higher credit levels in prosperous periods. However, it can happen that banks underestimate their credit risk, making the economic system more vulnerable when default materializes. In this case, we observe a negative correlation of 0.71 between aggregate production in time *t* and the leverage of firms in the previous time step.

**Figure 3:**
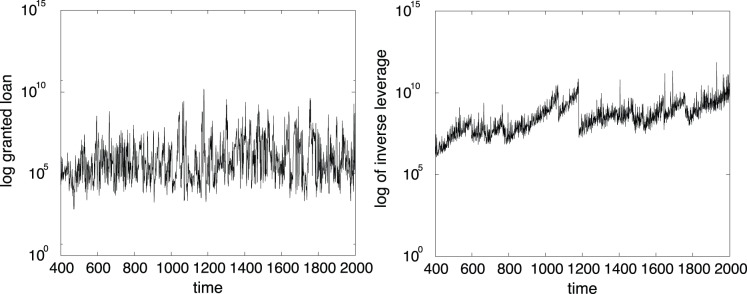
Time series of granted loan (left side) and the inverse of the firms’ leverage (right side).

Figure 3 shows time series of granted loan (left side) and the inverse of firms leverage. The balance sheet identity implies that firms can finance their capital stock by recurring either to net worth (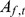) or to bank loans (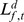), 
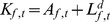
. From Eq (6) we can easily calculate firm equity 
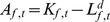
. So, the leverage is equal to 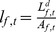. In the graph (3) (left side), we plot 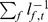. Comparing Figure 3 and Figure 1 (left), we observe that these three time series co-evolve. In particular, the simulated aggregate output suffers a severe crisis in 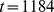, which is anticipated by a rapid increase in the financial fragility in the previous time steps (in fact the inverse of leverage decreases rapidly, as shown in Figure 3 (left)). Our findings support Minsky’s view. Expectations exceeding the actual demand are the main driving force behind over-leveraging and investing in riskier projects. When firms expect to be able to sell higher levels of output, they increase their loans. Banks, facing incomplete information about the true probability of good and bad outcomes, increase their borrowing to expand their balance sheet. This results in much higher defaults and financial instability once a bad state occurs (see [53]–[57] for empirical evidence).

Although companies in our model initially start with the same amount of capital and cash, trading generates a fat tail distribution of agents’ size, in accordance with the empirical evidence that, in real industrialized economies, market participants are very heterogeneous in dimension (see for example, [58]–[63]). Small and medium size firms -here we use firm production as proxy of firm size - dominate the economy. Large firms are relatively rare, but they represent a large part of total supply. When the firms size distribution is skewed, the mean firm size is larger than the median one, and both are larger than the modal firm size. Clearly, in this case the very notion of a representative firm is meaningless.

**Figure 4:**
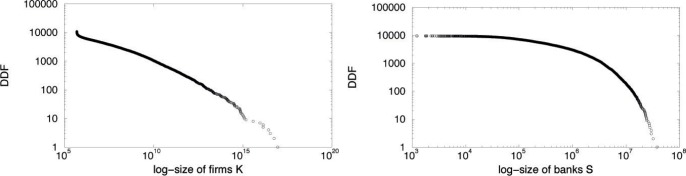
Decumulative distribution function of firm sizes (left side) and bank sizes (right side).

Figure 4 (left side) displays this evidence and the distribution is well fitted by a power law distribution 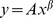, with intercept 12.19 and slope −0.23. The result is robust to the Kolmogorov-Smirnov test.

Our analysis on banks sizes (see Figure 4 (right)) reveals a similar skewed distribution. In this case, the Kolmogorov-Smirnov test is consistent with the null hypothesis of a lognormal distribution of bank sizes [64], [65].

### Default Cascades in the Interbank Market

In this section we explore the impact of the interbank market, in which each bank can be borrower and lender, at the same time, on the macroeconomic dynamics. In particular, we investigate the effect of credit risk and systemic risk on the aggregate fluctuations and on the dynamic of default cascades of banks.

Since the purpose of this exercise is to study the evolution of a self-contained system with a given initial number of banks, we exclude the possibility that failing banks would be replaced by new entrants.

**Figure 5:**
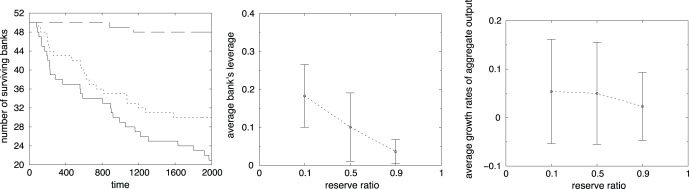
Time evolution of the number of surviving banks for different levels of reserve ratios: 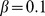 (solid line), 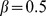 (dotted line) and 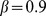 (long dashed line) (left side). Average bank’s leverage over time and simulation as a function of 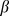 (center). Average output growth rate over time and simulation as a function of 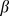 (right side).

The first question concerns the role of reserve requirements, reflected by the 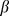 parameter in Eq (8). Figure 5 shows how different reserve ratios affect the fraction of surviving banks for the case of no interbank credit market (Higher 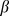 means higher reserves). As the reserve ratio 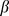 increases, the rate of bank failures clearly falls. This result is in line with other publications regarding the role of reserves (see Thurner et al. (2003) and Iori et al. (2006)). Obviously, increasing reserves contribute to the stability of individual banks, as shown by a lower value of average bank leverage (see center of Figure 5). However, increasing reserves reduces the output growth rate, since many firms do not get loans in the credit market (see right side of Figure 5).

We now analyze how different degrees of linkage in the interbank market affect the bankruptcy of financial institutions.

**Figure 6:**
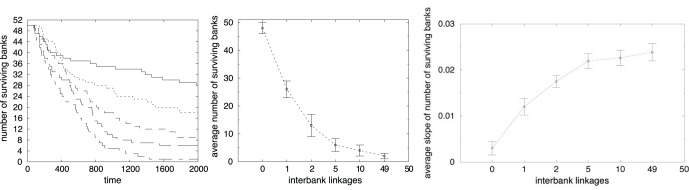
Time evolution of the number of surviving banks with 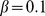 for different interbank linkages: 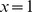 (solid line), 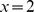 (dotted line), 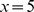 (dashed line), 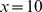 (long dashed line), 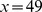 (dot-dashed line) (left side). Average number of surviving banks as a function of *x* (center). Average absolute slope of the curve representing the number of surviving banks (right side) as a function of *x*.

The left panel of Figure 6 displays the number of surviving banks as function of time, for various numbers *x* of financial institutions each bank randomly links with. By increasing linkage, the systemic risk raises in the sense that in any period, more banks fail. Indeed, with 100 percent linkage, the system collapses completely, analogously to a tragedy of the commons [66]. This result is further analyzed by Figure 6 (center), which shows the average number of surviving banks, over all times and all simulations as a function of the number of interbank linkages. While the earlier empirical literature on the systemic risk, in line with Allen and Gale’s result on the risk sharing role, found a very little evidence of global vulnerability [26], [67]–[69]. Strong evidence has been collected after the default of Lehman Brothers, showing that interbank linkages strongly impact systemic risk (see Battiston et al. (2012a), [70], Wagner (2010)) through a high probability of domino effects. So, in line with these new empirical and theoretical works, we find that the default of an agent may increase the systemic risk by increasing the connectivity.

Moreover, increasing *x*, not only the number of bankruptcies increases, but the time path of surviving banks also declines much more rapidly over time. This result is shown in the right panel of Figure 6, where we plot the average absolute slope of the number of surviving banks curve as a function of *x*. This graph provides a first evidence of contagious failures, that is periods in which many banks collapse together.

**Figure 7:**
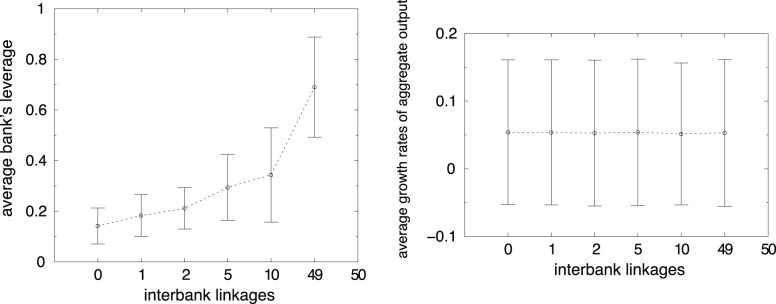
Average bank’s leverage (left side). Average output growth rate (right side), over time and simulation as a function of *x*.

In line with our hypothesis that a higher connectivity generates a higher systemic risk, not offset by a lower credit risk, Figure 7 shows, on the left, that the banks’ financial fragility increases with interbank linkages.

To understand if different linkages in the interbank market have some effect on the real economy, Figure 7 displays on the right hand side the average output growth rate as a function of *x* before bankruptcy cascades occur. One can immediately see that increasing the interbank connectivity has no effect on system growth. Companies have no benefits from a more strongly linked interbank market. In fact, it does not facilitate the granting of loans to enterprises, but it merely transfers liquidity among financial institutions.

**Figure 8:**
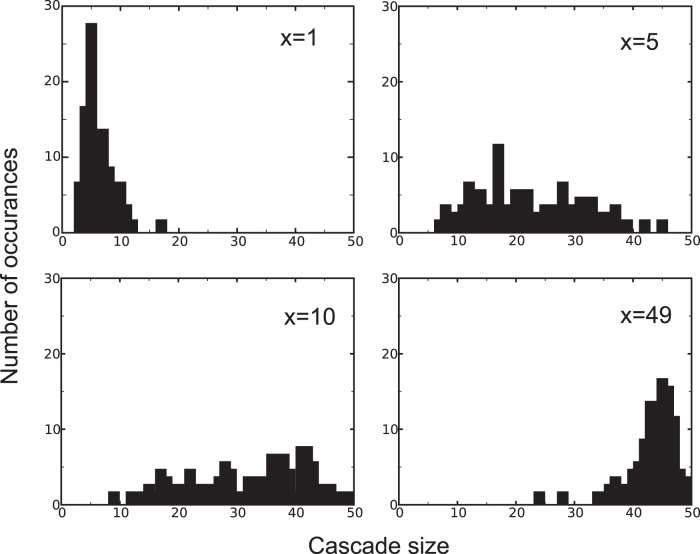
Size of the largest bankruptcy cascades, which are connected by bad debits for a bank market of size 50, determined from 100 simulations for interbank linkages of 1, 5, 10, and 49. A highly connected interbank market results in large cascades of bankruptcies.

We now turn to the issue of contagious failures. Banks are prone to default by bad debits of both the firm-bank credit market and the interbank market. To ensure that the higher number of bank bankruptcies in the case of a highly connected interbank market is not only the result of bad debits in the firm-bank market, but also is the result of more bad debits in the interbank market, we run the following experiment: we calculate the size of the largest connected component of the failed banks, which are connected by bad debits, in 100 simulations for each value of linkage in the interbank market (see Figure 8). As expected, a more inter-connected interbank market results in larger cascades of bankruptcies due the larger systemic risk.

**Figure 9:**
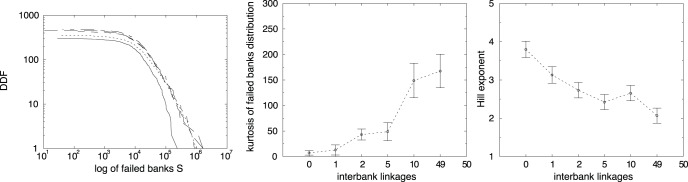
Decumulative distribution function of failed bank’s size *S*, for 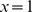 (solid line), 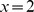 (dotted line), 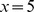 (dashed line), 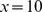 (long dashed line) and 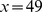 (dot-dashed line) (left side). Kurtosis (center) and Hill exponents (right side) of failed banks distribution as a function of *x*.

As for firms, we can infer that bankruptcy cascades depend on the size of failed banks -here we use bank liquidity *S* as proxy of bank size -. In fact, the distribution of failed banks for different interbank linkages is skewed (see left panel in Figure 9). Moreover, increasing the interbank connectivity creates fatter tails in the distribution of failed banks, as evidenced by a higher kurtosis (see center of Figure 9). A more precise measure of fat tails is provided by the Hill exponent. In the right panel of Figure 9, we plot the Hill exponent as a function of *x*. Empirically the tail exponent is found to take values between 2 and 4. Changing the parameters of the model our simulations generates values of the Hill exponent in the same range. When 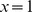, that is for low connectivity in the interbank market, the tail exponent is closer to the “normal” value of 4. However, increasing 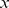, the model generates fatter tails.

### Conclusions

In this paper we have investigated systemic risk and the impact of sharing risk and in an interbank market. We have studied the agents’ financial fragility and the macroeconomic performance. The focus has been on how the emergent heterogeneity of market participants and the nature of their interconnectedness affect the trade off between mutual insurance and the potential for contagion.

We have shown that a higher banks connectivity not only increases the agent’s financial fragility, but also generates larger bankruptcy cascades due the larger systemic risk. Interestingly, high interbank linkages have no effect on economic output, even during boost/boom. The interbank market, in fact, just has a marginal effect on firms’ investments and on the granted loans. In contrast, higher bank reserve requirements stabilize the economic system, not only by decreasing financial fragility but also dampening avalanches. However, holding in reserve a larger percentage of banks’ equity affects the aggregate output growth by reducing credit to companies.

Our simulation results also indicate that heterogeneity contribute to instability. Although this result is strictly related to the dynamic of our model, other theoretical studies [10], [71] have shown that the possible emergence of contagion depends crucially on the degree of heterogeneity. Indeed, when the agents’ balance sheets are heterogeneous, banks are not uniformly exposed to their counterparty. Therefore, if the contagion is triggered by the failure of a big bank, which represents the highest source of exposure for its creditors, the situation is certainly worse than when agents are homogeneous. One policy implication is that interbank lending relationships should be restricted to banks that share similar liquidity characteristics. These results may be specific to our model, but they offer stimulating insights into the nature of contagion.

The main limitation of this study is that our model is fully demand-driven, i.e. firms can sell all the output that market exogenously can absorb at a fixed price. In a future paper, we will extend this analysis by including endogenous prices, which will allow us to investigate the demand side as well. Furthermore, we will introduce a more realistic mechanism of interbank linkages, by modeling network structures in an evolutionary way.
